# Renal Cell Carcinoma Associated with Xp11.2 Translocation/TFE3 Gene Fusion: A Rare Case Report with Review of the Literature

**DOI:** 10.1155/2013/810590

**Published:** 2013-12-22

**Authors:** Puneet Ahluwalia, Balagopal Nair, Ginil Kumar

**Affiliations:** Department of Urology, Amrita Institute of Medical Sciences, Kochi, Kerala 682041, India

## Abstract

*Introduction*. The recently recognized renal cell carcinomas associated with Xp11.2 translocations are rare tumors predominantly reported in children. Chromosome Xp11.2 translocation results in gene fusion related to transcription factor E3 (TFE3) that plays an important role in proliferation and survival. *Case Report*. Herein, we present two cases of a TFE3 translocation-associated RCC in young female adults, one detected incidentally and the other one presenting with gross hematuria. Tumor is characterized by immunohistochemistry and a literature review with optimal treatment regimen is presented. *Discussion*. Xp11.2 translocation RCCs in adult patients are associated with advanced stages, large tumors, and extracapsular disease and usually have an aggressive clinical course. *Conclusion*. In TFE3 RCC, the genetic background may not only contribute to tumorigenesis, but also determine the response to chemotherapy and targeted therapy. Therefore it is necessary to diagnose this tumor entity accurately. Because of the small number of *TFE3* gene fusion-related renal tumors described in the literature, the exact biologic behavior and impact of current treatment modalities remain to be uncertain.

## 1. Introduction

Renal cell carcinoma (RCC), the most common kidney cancer, constitutes approximately 2-3% of all cancers worldwide with 30% of patients presenting with metastasis. The prognosis of RCC varies according to the stage and histological grade. With technological improvements and genetic profiling, the classification for RCC has expanded. However, despite these changes in classification, 75% of epithelial renal tumors are still diagnosed as clear cell carcinoma and include a wide range of entities not yet characterized. Supporting this explanation, translocations involving the Xp11.2 locus were recently described in patients previously diagnosed with clear cell RCC.

**Figure 1:**
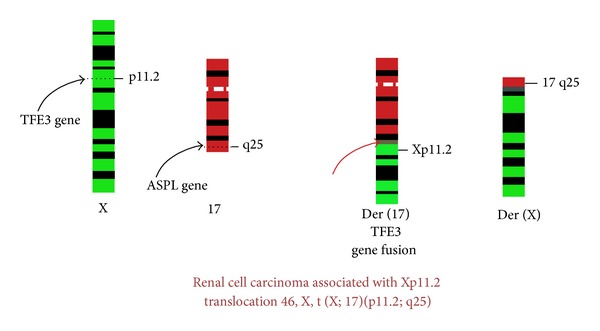
Renal cell carcinoma associated with Xp11.2 translocation 46, X, t(X; 17)(p11.2; q25).

Renal cell carcinoma associated with Xp11.2 translocations and *TFE3 *fusions (Xp11.2 TRCC) was only recognized as a distinct entity in 2004 WHO classification of kidney tumors [1]. Primarily described in the paediatric population (30% of RCC in children), RCC translocations have been reported recently in the adult population, with poorer prognosis [2]. These RCCs are characterized by various chromosome translocations, all of which involve a breakpoint at Xp11.2 as well as a fusion involving the TFE3 (transcription factor E3) gene (Figure 1).

## 2. Case History

**Figure 2:**
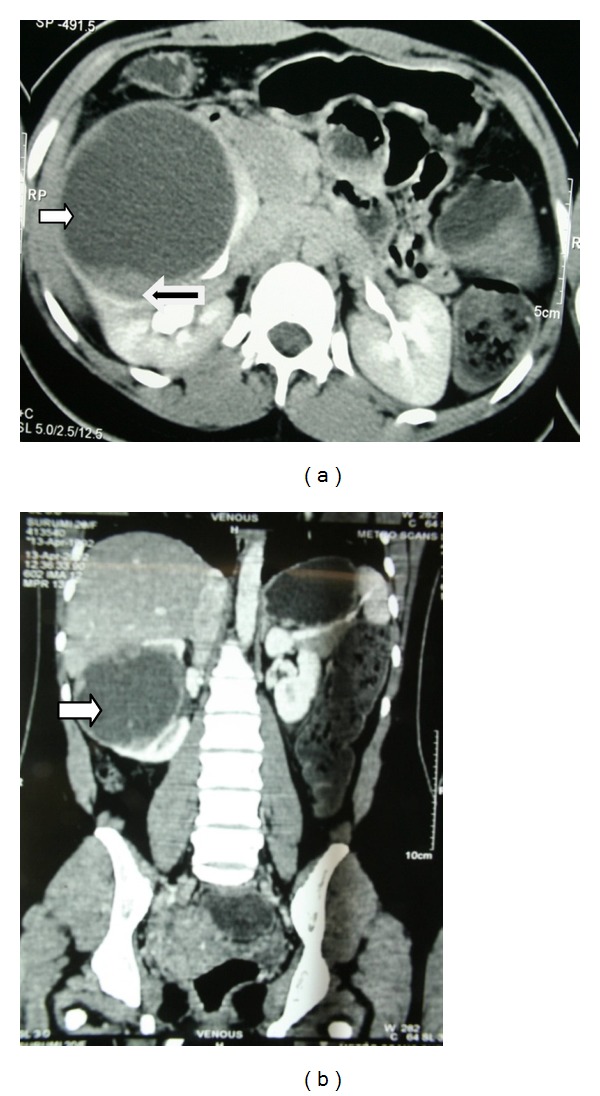
Contrast enhanced CT scan of the abdomen and pelvis in patient 1 with (a) axial section and (b) coronal section showing a large predominantly cystic (white arrow) right renal mass measuring 9 × 8 × 8 cm with solid areas (black arrow).

Two young female adult patients presented to us, one being 20 years old, incidentally found to have right renal mass (hereafter mentioned as patient 1), whereas the other patient, 17 years old, presented with history of total hematuria associated with clots and right flank pain (hereafter mentioned as patient 2). Contrast enhanced CT in both patients revealed findings consistent with renal neoplasm (Figure 2).

**Figure 3:**
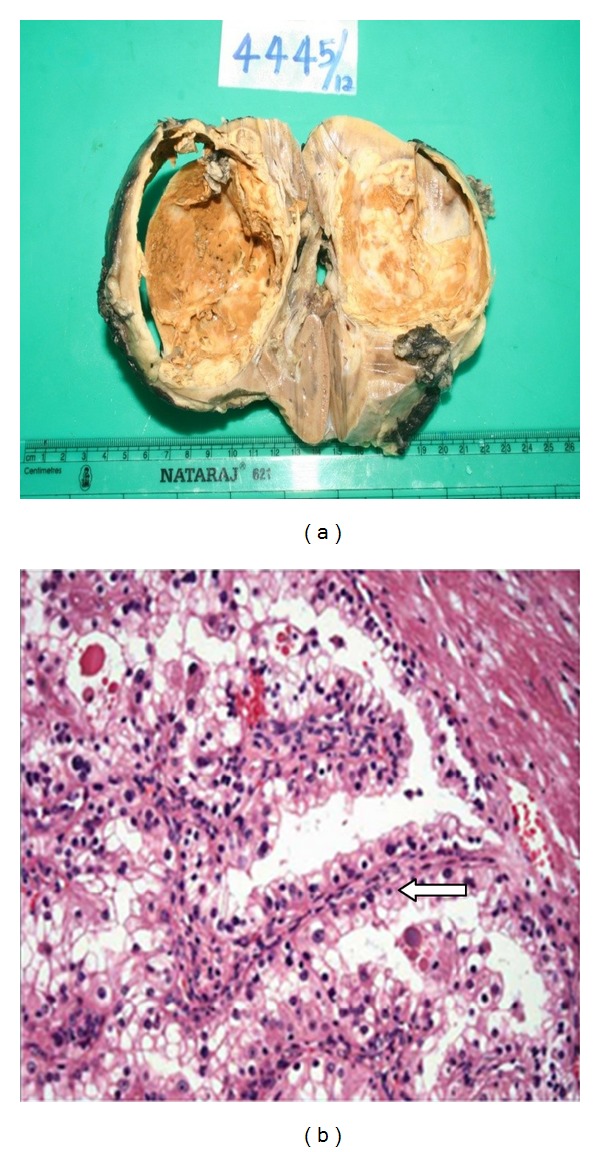
(a) Cut surface showing well encapsulated predominantly unilocular cystic lesion with grey brown solid areas with focal areas of haemorrhage measuring 7.5 × 9 × 7 cm in size. (b) Microscopically, cells are arranged in papillary pattern (arrow) with some areas showing alveolar and nesting pattern. Cells showed abundant clear to eosinophilic cytoplasm with prominent nucleoli.

After appropriate evaluation, both patients underwent right radical nephrectomy. Intraoperatively, the retroperitoneum demonstrated enlarged hilar lymph node mass in patient 1. Pathological examination of patient 1 showed well encapsulated predominantly unilocular cystic lesion with grey brown solid areas with focal areas of haemorrhage, measuring 7.5 × 9 × 7 cm (Figure 3(a)). Pathological findings in patient 2 revealed well defined encapsulated grey white to yellow white solid/cystic lesion in midpole of right kidney measuring 3 × 3 × 5 cm. None of the patients had any sinus fat infiltration.

**Figure 4:**
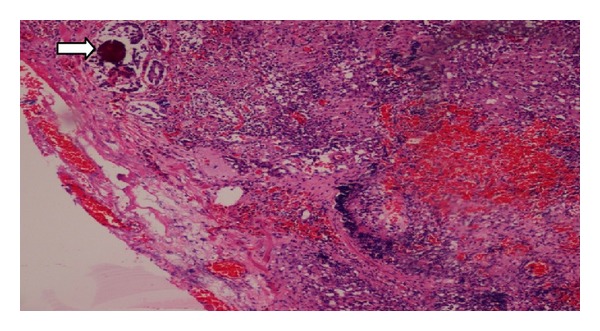
Hilar lymph node showing neoplastic infiltrate along with psammoma bodies (arrow).

Microscopically, the tumor was composed of cells arranged in papillary pattern with abundant clear to eosinophilic cytoplasm and prominent nucleoli (Figure 3(b)). Psammoma bodies were also seen frequently. Mitosis was seen occasionally. Hilar lymph node showed neoplastic infiltration in one of the patients (Figure 4). The differential diagnosis was between RCC with Xp11 translocation and papillary RCC type 2.

Immunohistochemistry was done and cells showed positivity for CD10, Vimentin, and EMA but were negative for CK7. These features were suggestive of renal cell carcinoma with Xp11 translocation. TFE3 gene mutation study was further done at John Hopkins Hospital, Baltimore, where immunostains showed tumor cells to be positive for TFE3 but negative for cathepsin K. FISH analysis showed 55% and 36.7% cells to have split TFE3 signal in patient 1 and patient 2, respectively, supporting a diagnosis of translocation renal cell carcinoma.  Table 1 summarizes the findings in both patients. Both patients have completed approximately one year of followup with serial physical examination, chest X-ray, and laboratory tests and were found to be disease-free. MRI abdomen was found to be normal with no residual disease or any evidence of metastasis.

## 3. Discussion

Xp11.2 translocation RCCs in adult patients may be associated with advanced stages, large tumors, and extracapsular disease and may present with metastatic disease with possible poor prognosis. Female predominance is seen in adults, as seen in our patients also. In young patients, Xp11.2 RCC should be suspected if prominent lymph node metastases are present as seen in one of our patients.

Grossly, these tumors usually have a variegated appearance. Histologically these tumors may resemble clear cell RCC (CCRCC), papillary RCC (PRCC), and clear cell papillary RCC (CCPRCC), a recently recognized entity. Immunohistochemical markers are helpful in the differential diagnoses (Table 2). If findings are equivocal on histology and IHC staining, FISH assay and RT-PCR are useful confirmatory tests.

In unclassified cases of RCC, adverse clinicopathological parameters are associated with positive expression of transcription factor E3 (TFE3) [3]. Studies have shown that the 5-year cancer-specific survival rate for TFE3-positive patients was 15.6% as compared to 87.5% for TFE3-negative patients [3]. TFE3 positivity in RCC was significantly associated with shorter cancer specific survival (*P* < 0.001).

The current management of Xp11.2 RCC is similar to conventional RCC. For localized Xp11.2 RCC including patients with positive regional lymph nodes, surgery is the treatment of choice. For patients with hematogenous metastases, the current options are immunotherapy using cytokines, such as interleukin 2 (IL-2) and interferon alpha (IFN*α*), and multikinase inhibitors. Malouf et al. analyzed the benefit of targeted therapy (VEGFR targeted agents and/or mTOR inhibitors) in patients with Xp11 translocation/TFE3 fusion gene metastatic RCC and found better response in terms of median progression-free survival (PFS) as compared to PFS of 2 months when receiving a cytokine-based regimen [4].

The grim clinical outcome associated with Xp11.2 RCC warrants early detection, accurate diagnosis, and close followup. Till recently no surveillance algorithm for Xp11 TRCC after radical nephrectomy was developed. Recently Zachary et al. proposed classification of these tumors as high risk and recommended aggressive followup with regular physical examination, laboratory tests, CT chest, and CT abdomen up to 10 years of duration [5]. Furthermore, because Xp11 TRCC is often diagnosed in young adults, they advocated lifelong followup with yearly history, physical examination, and laboratory tests and chest and/or abdominal imaging as deemed clinically necessary after completing the 10-year regimen.

## Figures and Tables

**Table 1:** Summarizing the findings in both patients with Xp11 translocation RCC.

	Patient 1	Patient 2
Age	20 years	17 years
Presenting symptom	Incidentally detected	Total hematuria with clots Right flank pain
Physical examination	Palpable mass in right hypochondrium	Unremarkable
USG	8 cm cyst in anteromedial portion of right kidney/few internal septa	Hyperechoic lesion in the midpole region of right kidney measuring 4 × 3 cm in size
CECT	(i) Large 9 × 8 × 8 cm partially exophytic cystic mass arising from anterior interpolar region of right kidney with few small enhancing mural nodules in inner margin of cyst wall with prominent solid areas in medial wall (ii) No lymphadenopathy (iii) Renal vein and IVC normal	(i) Heterogeneously enhancing, well defined rounded lesion in the posterior aspect of right kidney near lower pole measuring 5 × 4 × 4 cm in size with solid and cystic areas, spanning the cortex and medulla with minimal bulging into the sinus region. (ii) No lymphadenopathy (iii) Renal vein and IVC normal
Surgery	Right open radical nephrectomy. Enlarged hilar lymph node seen	Right laparoscopic radical nephrectomy
HPE	(i) RCC with Xp translocation-like features (ii) Neoplastic infiltrate in hilar lymph node (iii) Stage: pT2N1Mx	(i) RCC with Xp translocation-like features (ii) No evidence of infiltration to perinephric or renal sinus fat (iii) Stage: pT1bNxMx
Immunostain	Positive for CD10, Vimentin, and EMA but negative for CK7.	Positive for CD10, Vimentin, and EMA but negative for CK7.
TFE3 gene mutation study	TFE3 positivity with FISH analysis showing 55% cells to have split TFE3 signal.	TFE3 positivity with FISH analysis showing 36.7% cells to have split TFE3 signal supporting diagnosis of Xp11 translocation RCC.

**Table 2:** The immunostain profiles of Xp11.2 RCC and its close mimickers.

	Xp11.2 RCC	CCRCC	PRCC	CCPRCC
TFE3	+	−	−	−
Cathepsin K	+	−	−	−
CK7	−	−	+	+
Vimentin	−	+	−	− Or focal +
AMACR	+	−	+	−
CD10	+	+	+	− Or focal +
CA9	−	+	−	+
